# Supplementary material for: Mental Health during the COVID-19 Lockdown over the Christmas Period in Austria and the Effects of Sociodemographic and Lifestyle Factors
Source: Int J Environ Res Public Health. 2021 Apr 1;18(7):3679. doi: 10.3390/ijerph18073679 (PMC8036255; doi:10.3390/ijerph18073679)
Supplement: Supplementary file 1 [file ijerph-18-03679-s001.pdf]

Supplementary Materials

Table S1: Categories with reduced quota as compared to a representative sample.

	Final %	Representative %	% Difference
Male 18-24	4.19	5.5	1.31
Male 25-34	8.64	9.13	0.49
Female 45-54	10.63	11.06	0.43
Female 65+	5.58	6.56	0.98
Vienna	22.99	23.44	0.45
Upper Austria	15.08	16.69	1.6
Salzburg	5.25	6.31	1.06
Tyrol	7.57	8.5	0.93
Vorarlberg	3.46	4.44	0.98
High Education	43.59	53.06	9.47

Below the significant results from the Bonferonni-corrected post-hoc analyses for age, marital status, income and physical activity are reported.

Table S2. Significant results of Bonferroni corrected post-hoc-tests for age groups.

WHOQOL-BREF-psychological domain

18 – 24 vs. 35-44 ($p = .01$), vs. 45-54 ($p = .02$), vs. 55 – 64 ($p < .001$) and vs. 65+ ($p < .001$)

25 – 34 vs. 55 – 64 ($p = .03$) and vs. 65+ ($p < .001$)

35 – 44 vs. 65+ ($p = .05$)

45 – 54 vs. 65+ ($p = .02$)

WHO-5

18 – 24 vs. 35-44 ($p = .04$), vs. 45-54 ($p = 0.03$), vs. 55 – 64 ($p < .001$) and vs. 65+ ($p < .001$)

25 – 34 vs. 65+ ($p < .001$)

35 – 44 vs. 65+ ($p = .008$)

45 – 54 vs. 65+ ($p < .001$)

55 – 64 vs. 65+ ($p = .05$)

PSS-10

18 – 24 vs. 35 – 44 ($p < .001$), vs. 45 – 54 ($p < .001$), vs. 55 – 64 ($p < .001$), and vs. 65+ ($p < .001$)

25 – 34 vs. 45 – 54 ($p = .03$) vs. 55 – 64 ($p < .001$) and vs. 65+ ($p < .001$)

35 – 44 vs. 55 – 64 ($p = .01$) and vs. 65+ ($p < .001$)

45 – 54 vs. 65+ (p < .001)
55 – 64 vs. 65+ (p = .02)
ISI
18 – 24 vs. 55 – 64 (p = .004) and vs. 65 (p < .001)
25 – 34 vs. 65+ (p < .001)
35 – 44 vs. 65+ (p < .001)
45 – 54 vs. 65+ (p < .001)
GAD-7
18 – 24 vs. 35 – 44 (p < .001), vs. 45 – 54 (p < .001) vs. 55 – 64 (p < .001), and vs. 65+ (p < .001)
25 – 34 vs. 55 – 64 (p = .003) and vs. 65+ (p < .001)
35 – 44 vs. 65+ (p < .001)
45 – 54 vs. 65+ (p = .001)
55 – 64 vs. 65+ (p = .01)
PHQ-9
18 – 24 vs. 25 – 34 (p = .006), vs. 35 – 44 (p <.001), vs. 45 – 54 (p < .001), vs. 55 – 64 (p < .001), and vs. 65+ (p < .001)
25 – 34 vs. 35 – 44 (p = .006), vs. 55 – 64 (p < .001), and vs. 65+ (p < .001)
35 – 44 vs. 65+ (p < .001)
45 – 54 vs. 65+ (p < .001)
55 – 64 vs. 65+ (p = .02)

Table S3: Significant results of Bonferroni corrected post-hoc-tests for marital status.

WHOQOL-BREF-psychological domain
single vs. married (p < .001) and vs. cohabiting (p = .02)
separated vs. married (p = .003)

WHO-5
single vs. married ($p < .001$) and vs. widowed ($p = .03$)
separated vs. married ($p < .001$) and vs. widowed ($p < .001$)
PSS-10
single vs. married ($p < .001$) and vs. widowed ($p = .02$)
separated vs. married ($p = .01$), vs. widowed ($p < .001$) and vs. divorced ($p = .001$)
married vs. cohabiting ($p = .03$)
ISI
separated vs. married ($p = .04$)
GAD-7
single vs. married ($p < .001$)
separated vs. married ($p = .03$)
PHQ-9
single vs. married ($p < .001$)
separated vs. married ($p = .007$)
married vs. cohabiting ($p = .04$)

Table S4: Significant results of Bonferroni corrected post-hoc-tests for monthly net income.

WHOQOL-BREF-psychological domain
<1000 vs. 2-3000 ($p < .001$), vs. 3-4000 ($p < .001$), and vs. >4000 ($p < .001$)
1-2000 vs. 3-4000 ($p < .001$), vs. >4000 ($p < .001$)
2-3000 vs. >4000 ($p < .001$)
3-4000 vs. >4000 ($p = .03$)
WHO-5

<1000 vs. 2-3000 (p = .006), vs. 3-4000 (p = .002), and vs. >4000 (p < .001)
1-2000 vs. >4000 (p < .001)
2-3000 vs. >4000 (p < .01)
PSS-10
<1000 vs. 2-3000 (p < .02), vs. 3-4000 (p = .001), and vs. >4000 (p < .001)
1-2000 vs. 3-4000 (p = .03), vs. >4000 (p < .001)
2-3000 vs. >4000 (p = .005)
ISI
<1000 vs. >4000 (p < .001)
1-2000 vs. >4000 (p < .001)
2-3000 vs. >4000 (p < .001)
3-4000 vs. >4000 (p = .005)
GAD-7
<1000 vs. 2-3000 (p = .006), vs. 3-4000 (p = .002), and vs. >4000 (p < .001)
1-2000 vs. >4000 (p < .001)
2-3000 vs. >4000 (p = .007)
PHQ-9
<1000 vs. 1-2000 (p = .03) vs. 2-3000 (p < .001), vs. 3-4000 (p < .001), and vs. >4000 (p < .001)
1-2000 vs. 3-4000 (p = .03), vs. >4000 (p < .001)
2-3000 vs. >4000 (p = .001)

Table S5: Significant results of Bonferroni corrected post-hoc-tests for number of days of physical activity per week.

WHOQOL-BREF-psychological domain
0 vs. 1 (p = .003), vs. 2 (p < .001), vs. 3 (p < .001) and vs. >4 (p < .001)

1 vs. >4 (p = .007)<br>2 vs. >4 (p = .02)
WHO-5<br>0 vs. 1 (p = .008), vs. 2 (p < .001), vs. 3 (p < .001) and vs. >4 (p < .001)<br>1 vs. >4 (p < .001)<br>2 vs. >4 (p = .005)
PSS-10<br>0 vs. 2 (p = .005), vs. 3 (p < .001) and vs. >4 (p < .001)
ISI<br>0 vs. >4 (p = .03)
GAD-7<br>0 vs. 3 (p < .01) and vs. >4 (p < .001)
PHQ-9<br>0 vs. 2 (p = .04), vs. 3 (p < .001) and vs. >4 (p < .001)<br>1 vs. >4 (p = .04)<br>2 vs. >4 (p = .03)

The results of the current survey were compared with those from other representative surveys conducted in 2019 and during the April 2020 lockdown. All three surveys included PHQ-8 scores for depressive symptoms and WHOQOL-BREF scores for quality of life in the psychological domain.

Table S6: Comparisons of PHQ-8 depression and WHOQOL-BREF quality of life in 2019, the April lockdown and the December/January lockdown.

		**Sample**			**Statistic**
		ATHIS 2019 N=7417876	April lockdown N=1005	Dec/Jan lockdown N=1505	
**PHQ-8 Cut-off N (%)**	≥ 10	416844 (5.6%)	200 (19.9%)	378 (25.1%)	χ^2 (2)= 1463.2, p<0.001)
**PHQ-8 score**	M (SD)	2.76 (3.44)	5.93 (5.0)	6.54 (5.48)	F (2, 7420382) = 1334.7, p<0.001
**WHOQOL psychological domain**	M (SD)	79.74 (15.06)	69.83 (18.7)	68.89 (18.66)	F (2, 7420382) = 607.56, p<0.001)

Table S7: Percentage of people with moderate depression, mean depression score and mean quality of life score in 2018/19, April 2020 and December 2020/January 2021 according to gender. The changes between pre-pandemic and December 2020/January 2021 are also shown.

		**2018/2019**		**April lockdown**		**Dec/Jan lockdown**	
		Male	Female	Male	Female	Male	Female
**Moderate depression (%)**	PHQ-8 ≥ 10	4.4%	6.8%	15.6%	23.8%	19.4% (+15%)	30.6% (+23.8%)
**Depression score (mean)**	PHQ-8	2.35	3.15	5.15	6.63	5.65 (+3.3)	7.41 (+4.3)
**Quality of life (mean)**	WHOQOL PSYCH	81.11	78.43	72.75	67.21	71.87 (-9.24%)	66.0 (-12.43%)

The results from the current survey were also compared with results from a different, but also representative, sample surveyed during the first lockdown in April in Austria. The comparisons of the percentage of people over the cut-off scores for depression, anxiety and insomnia according to age group and family status are presented below.

Table S8: Percentage of people over the cut-off for moderate depression, anxiety and insomnia by age category in the first and second lockdowns.

		**April lockdown**					
		18-24	25-34	35-44	45-54	55-64	65+
**PHQ-9 score (%)**	≥ 10	32.2	33.7	20.0	19.8	16.0	5.3
**GAD-7 score (%)**	≥ 10	22.0	27.1	20.0	19.8	16.6	6.8
**ISI score (%)**	≥ 15	18.6	18.1	14.1	20.3	16.0	4.5
		**December/January lockdown**					
		18-24	25-34	35-44	45-54	55-64	65+
**PHQ-9 score (%)**	≥ 10	50.3	30.5	25.3	24.5	22.1	11.8
**Lockdown comparison (PHQ-9)**		t(269)=3.03, p<0.01)	t(443)=-0.72, p>0.05)	t(472)=1.32, p>0.05	t(546)=1.3, p>0.05	t(451)=1.59, p>0.05	t(317)=2.02, p<0.05
**GAD-7 score (%)**	≥ 10	34.6	28.3	23.2	24.9	18.8	10.2
**ISI score (%)**	≥ 15	25.5	18.3	20.8	21.2	15.1	11.3

Table S9: Percentage of people over the cut-off for moderate depression, anxiety and insomnia by marital status in the first and second lockdowns.

		April lockdown					
		single	separated	divorced	cohabiting	married	widowed
**PHQ-9 score (%)**	≥ 10	26.9	25.0	25.0	23.6	14.8	23.5
**GAD-7 score (%)**	≥ 10	21.4	18.8	27.9	19.0	15.8	23.5
**ISI score (%)**	≥ 15	17.1	18.8	22.1	15.3	14.6	0
		**December/January lockdown**					
		single	separated	divorced	cohabiting	married	widowed
**PHQ-9 score (%)**	≥ 10	35.1	46.5	30.5	24.9	18.5	20.6
**GAD-7 score (%)**	≥ 10	27.1	34.9	23.8	24.2	18.9	17.6
**ISI score (%)**	≥ 15	20.6	37.2	21.0	16.9	16.6	14.7
